# Burning our way to a cure?

**DOI:** 10.1016/j.igie.2024.02.001

**Published:** 2024-02-06

**Authors:** Henry Kyunghoon Shin, Linda S. Lee

**Affiliations:** 1 STARmed Co., Ltd., Goyang-si, South Korea; 2 Division of Gastroenterology, Hepatology and Endoscopy, Brigham and Women’s Hospital, Boston, Massachusetts, USA

## Editor’s Introduction

Radiofrequency ablation (RFA) was first used by surgeon Martin Kirschner in the early 1930s to treat trigeminal neuralgia.^1^ Since then, percutaneous and endoscopic applications of RFA have blossomed for both benign and malignant indications. Robert A. Ganz, Roger Stern, and Brian Zelickson developed the balloon-based RFA system to treat esophageal metaplasia and dysplasia in 1999, and it has become standard treatment to manage Barrett’s esophagus with dysplasia in a safe, durable manner.^2^ Pancreaticobiliary indications for RFA are still in the early stages of development and understanding. Photodynamic therapy revealed improved biliary stent patency and mortality but was limited by phototoxicity and cumbersome procedural experience, the latter having been overcome by biliary RFA probes. In the United States, the Habib EndoHPB probe (Boston Scientific, Marlborough, Mass, USA) received U.S. Food and Drug Administration (FDA) 510(k) clearance in July 2009, and this device is generator agnostic although it requires specific settings to avoid thermal injury with the newest VIO 3 (Erbe Elektromedizin GmbH, Tübingen, Germany). Randomized trials with this device have reported discrepant findings over stent patency in RFA-treated patients while some studies suggested improved survival in patients with cholangiocarcinoma.^3^, ^4^, ^5^, ^6^, ^7^ The ELRA catheter of STARmed Co, Ltd (Goyang-si, South Korea) for biliary RFA treatment is unique as it is paired with a dedicated generator (VIVA Combo RF generator; STARmed Co, Ltd) that measures temperature, impedance, time, and wattage with safety mechanisms to prevent thermal injury. A pilot randomized trial confirmed the safety of ELRA and suggested decreased premature occlusion of plastic stents.^8^ This received FDA 510(k) clearance in 2019 while the EUSRA electrode (STARmed Co, Ltd) received clearance in 2018. The latter is the only device currently available in the United States for EUS-guided RFA of pancreatic lesions. An increasing body of literature suggests efficacy of EUS-guided RFA for pancreatic neuroendocrine tumors, as well as potential treatment of pancreatic ductal adenocarcinomas and pancreatic cystic lesions.

I am delighted to discuss with Mr Henry Kyunghoon Shin, President and Chief Executive Officer of STARmed Co, Ltd, the development of their unique RFA devices. Henry is an accomplished Chief Executive Officer with extensive experience in the medical device industry. Leading STARmed Co, Ltd since 2009, he has a proven track record in international sales and commercialization of medical technologies. Henry has been recognized with several awards for his contributions to the industry and economic development. He has successfully filed numerous patents, significantly contributing to his company's global market expansion. Henry is fluent in English and Korean, with a professional background marked by strategic leadership and innovation in the medical business sector.

Section Editor: Linda S. Lee, MD

**Figure 1:**
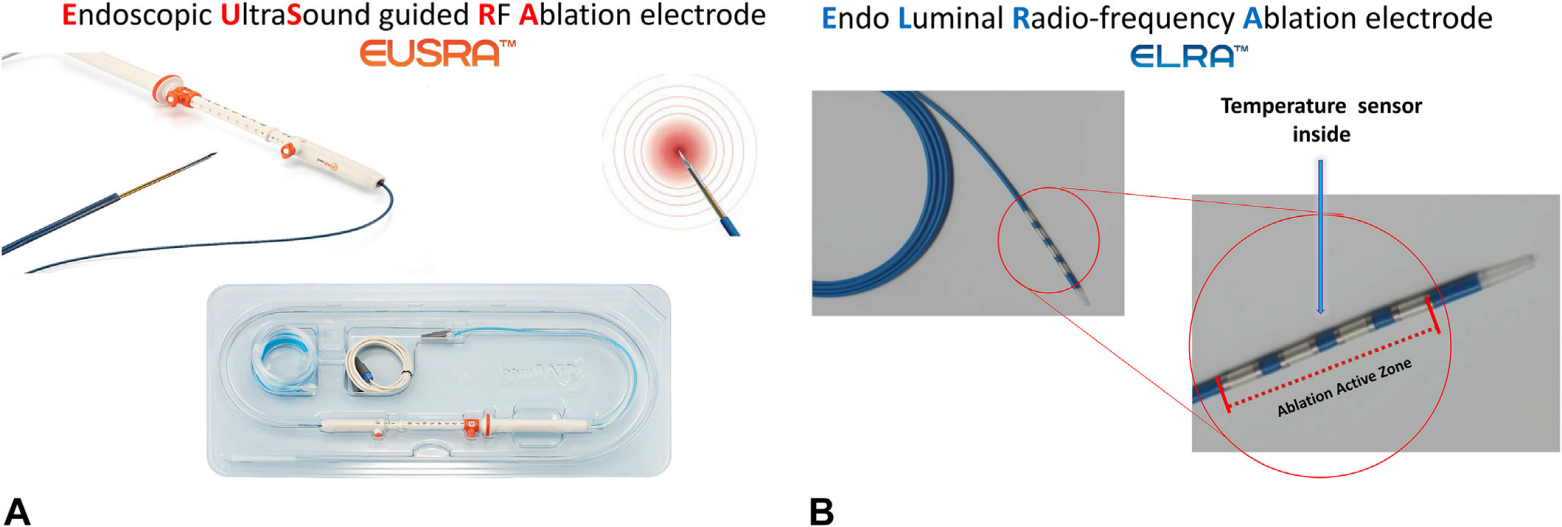
**A,** STARmed EUS-guided radiofrequency ablation needle (EUSRA). **B,** STARmed biliary radiofrequency ablation probe (ELRA).

**Linda S. Lee (LSL): Thank you very much for making time with your incredibly busy schedule to discuss the evolution of RFA in gastroenterology. Would you discuss how this idea came about and became a focus for your company, STARmed?** (Fig. 1)

**Henry Shin (HS):** Our company, STARmed, has specialized in RFA, accumulating significant technological expertise and clinical experience in liver RFA and thyroid RFA. While there were excellent energy-based devices in gastroenterology, there was a lack of treatment devices considering the location and shape of tumors. With the advancement of EUS, the need for a device that effectively treats tumors, especially in the pancreas, led to the development of EUSRA, an EUS-guided ablation device. ELRA was developed to address challenges in biliary stent usage, aiming to directly treat bile duct tumors and delay tumor ingrowth, requiring temperature-based precision control technology for ablation.

**Figure 2:**
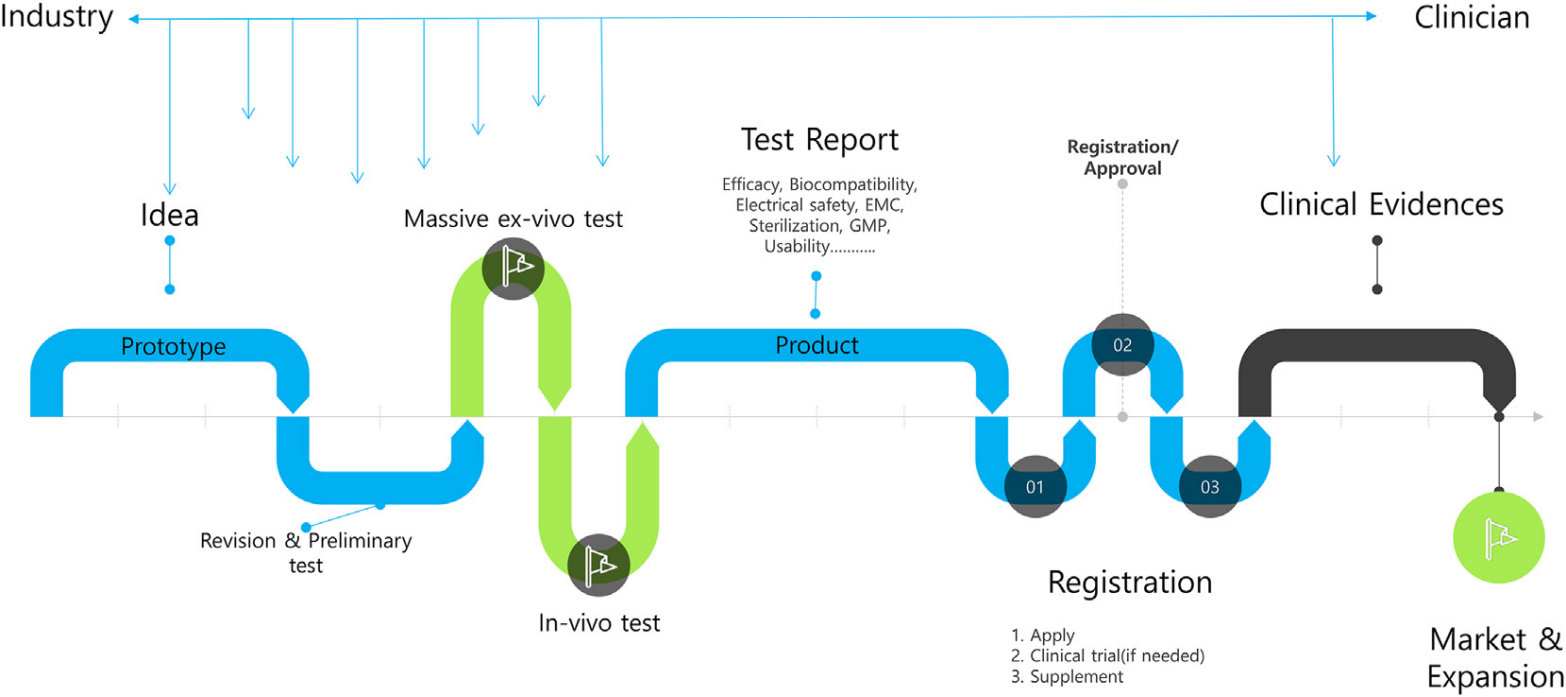
Key steps during the development of a radiofrequency ablation system. *ECM*, Electromagnetic compatibility; *GMP*, Good Manufacturing Practice.

**LSL: Would you discuss the key steps in taking the idea of a dedicated RFA system to something we can actually use in people? (** Fig. 2 **)**

**HS:** Industry or the clinician can come up with an idea. However, the gap between developers and actual users in the medical device industry is larger than in any other industry. Therefore, even if the idea was created first by the industry, it is very important to collaborate with clinicians to find efficient usage methods and points to improve.

In the case of these products, we started with the idea that the electrode used in conventional US-guided percutaneous RFA can also be applied to endoscopic-guided methods. We designed an electrode that is compatible with endoscopes and an RF output pattern that is suitable for endoscopic electrodes.

After producing a prototype that implemented the idea, we verified the basic performance through ex vivo experiments. We then collaborated with clinicians to review usability, conducted continuous improvement and verification, and identified the final improvement points through animal experiments.

We were also selected for support through the Korean Health Technology R and D project, Ministry of Health and Welfare, Republic of Korea. After obtaining domestic medical device approval, we continued to collaborate with clinicians to secure clinical evidence data through clinical research support programs.

**LSL: What were the challenges you faced as your company developed this, and how did STARmed overcome these challenges?**

**HS:** The first challenge we faced when we started developing the product was that the gastroenterology doctors who would be performing the procedure were not familiar with RFA. It took a lot of time to explain and understand the principles of coagulation, the need for internal water cooling, and the need for a temperature sensor.

The most difficult part was that because it was a product developed for a new procedure, there was very limited scientific and clinical information that could be directly benchmarked. It took a very long time and effort to realize our idea safely and efficiently in the actual clinical setting. Fortunately, thanks to the many doctors who highly valued the potential of our technology, they continuously participated in experiments and gave us advice, and we were able to overcome the limitations of a start-up company. Through this, we were able to develop and improve the product and reach this point.

**Figure 3:**
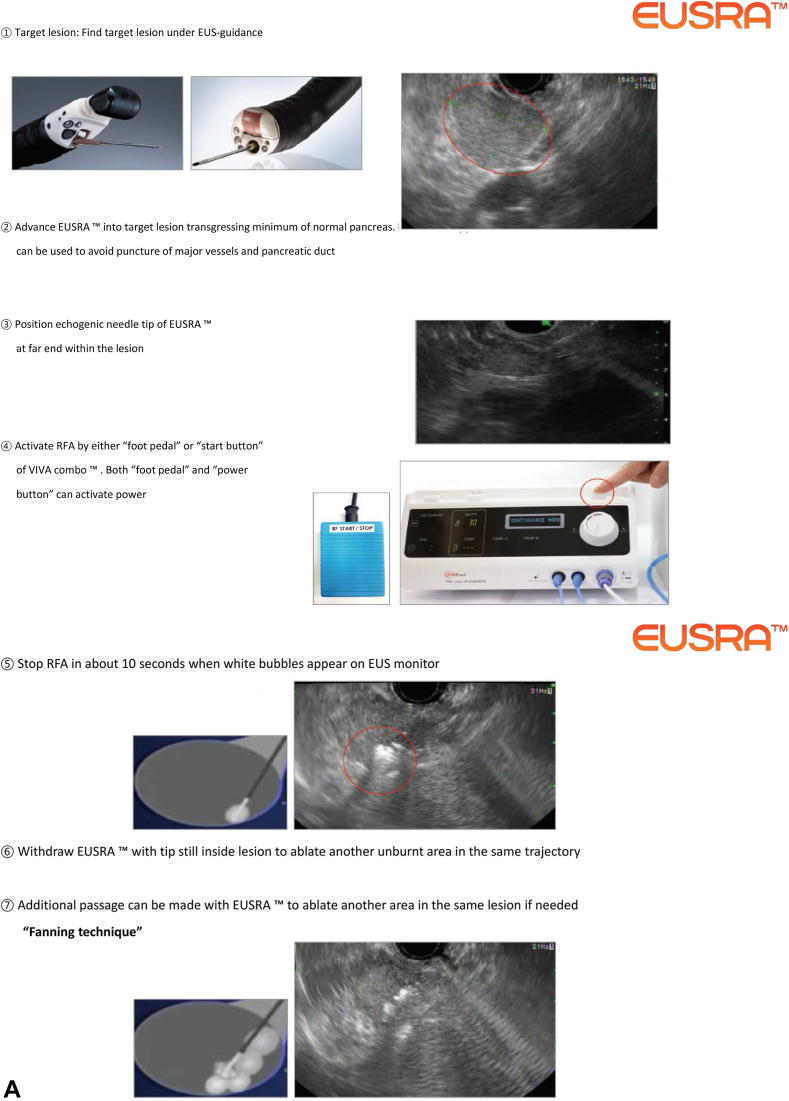

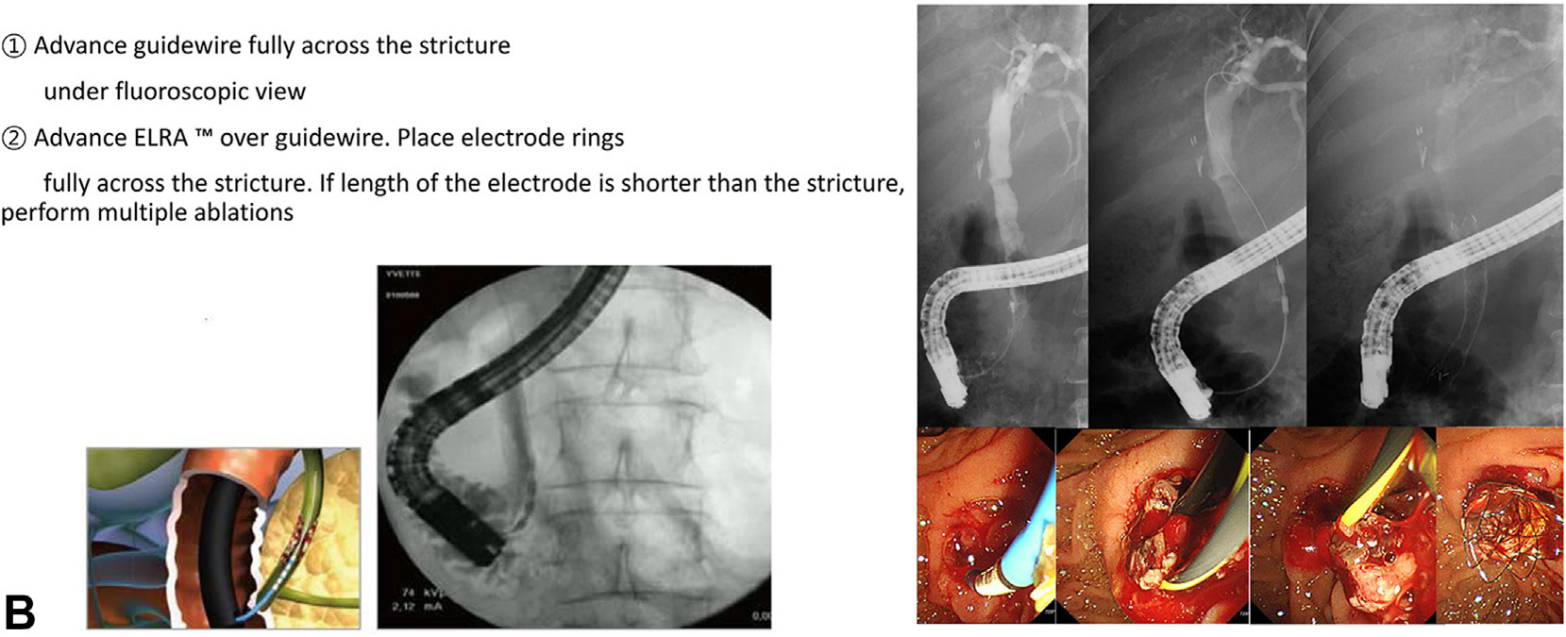
**A,** EUS-guided radiofrequency ablation (RFA) procedure. **B,** Biliary radiofrequency ablation procedure with ELRA.

**LSL: As you know, there have been other RFA devices both for the bile duct and pancreatic lesions that are no longer on the market. How has your product endured? (** Fig. 3 **)**

**HS:** Through numerous trial and error, we have confirmed that precise coagulation through the optimization of the generator and applicator is the most important factor in treatment performance. In EUSRA, we applied an internal cooling system and implemented a stable and predictable coagulation pattern through impedance feedback. In ELRA, we were able to achieve precise coagulation through a temperature sensor, improving treatment performance compared with other companies while minimizing adverse events.

**LSL: How does the approval process for medical devices work in South Korea compared with the FDA in the United States?**

**HS:** First I will outline the process in South Korea.

1. Regulatory Body: In South Korea, the Ministry of Food and Drug Safety, formerly known as the Korea Food and Drug Administration, is the primary regulatory body.
2. Approval Process:
  • Premarket Notification: For lower risk devices (Class I), a simpler Premarket Notification is sufficient.
  • Premarket Approval (PMA): Higher risk devices (Class II and III) require a more comprehensive PMA.
  • Clinical Trials: For certain high-risk devices, clinical trials in South Korea might be required.
  • Quality Management System: Manufacturers must comply with the Korea Good Manufacturing Practice standards.
3. Documentation: Manufacturers need to provide detailed documentation, including technical files and clinical data.
4. Local Representation: Foreign manufacturers must appoint a Korean License Holder to interact with the Ministry of Food and Drug Safety.

This is the process in the United States:

1. Regulatory Body: The FDA oversees medical device regulation in the United States.
2. Approval Process:
  • 510(k) Clearance: Most medium-risk devices (Class II) are subject to 510(k) clearance, in which the device is compared versus a legally marketed predicate device.
  • PMA: High-risk devices (Class III) require PMA, which involves a thorough review of clinical and scientific evidence to ensure safety and effectiveness.
  • De Novo Classification: For novel devices without a comparable predicate, the De Novo pathway is an option.
3. Clinical Trials: The FDA may require clinical trials, especially for high-risk devices.
4. Quality System Regulation: Manufacturers must adhere to the FDA’s Quality System Regulation, which is similar to international standards such as ISO 13485.
5. Documentation: Extensive documentation, including preclinical and clinical data, is required.
6. Registration and Listing: Manufacturers and their devices must be registered with the FDA.

These are the key differences between South Korea and the United States.

• Regulatory Pathways: The U.S. has distinct pathways such as 510(k), PMA, and De Novo, whereas South Korea primarily differentiates between Premarket Notification and PMA.
• Clinical Trial Requirements: The FDA often has more stringent requirements for clinical data, especially for novel devices.
• Quality Standards: Although both require compliance with quality standards, South Korea has specific Korea Good Manufacturing Practice standards.
• Local Representation: South Korea requires foreign manufacturers to have a Korean License Holder, whereas in the United States, foreign companies must appoint a U.S. agent for FDA communication.

**LSL: What were the particular challenges in having this product approved by the FDA in the United States?**

**HS:** The scope of the data required by the FDA in 2018 was wider or different from the scope required by the Korean Ministry of Food and Drug Safety when we first received approval in 2013. Additional safety data on multiple human tissues, as well as additional reports on environmental conditions in clinical procedures, were required. We understood that the FDA did not request any special standards but rather that the standards for safety had increased over time. We focused our efforts to submit the data in a short period of time.

**Figure 4:**
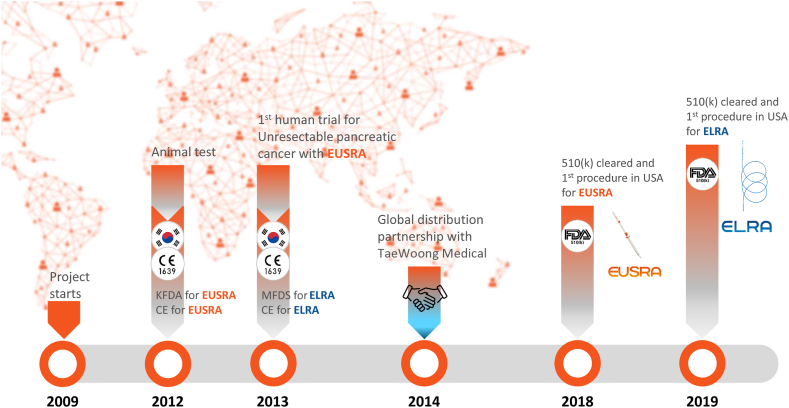
Timeline of key events during the development of EUSRA and ELRA. *KFDA*, Korea Food and Drug Administration.

**LSL: Would you discuss what your strategy has been in introducing this product globally and the relationship with TaeWoong? (** Fig. 4 **)**

**HS:** TaeWoong Medical is our sister company, and STARmed was spun off from TaeWoong Medical in 2009 to focus more on the RFA business.

However, EUSRA and ELRA are products used in the gastroenterology field, which is TaeWoong Medical’s strong point. To introduce these products to the world, we signed a global distribution agreement with TaeWoong Medical in 2014.

Because the procedure and the product were not familiar to users, we focused on educating them and giving them hands-on experience. We also focused on conducting regular education workshops and training courses through our collaboration with TaeWoong Medical. We also supported the continued publication of high-quality clinical data.

**LSL: Although available in the United States, the technology is not widely used. Why do you think this is, and what are the strategies to expand use of the RFA system?**

**HS:** The procedure is not widely performed and requires a new approach and technique. In addition, EUSRA requires EUS skills. It took time to create clinical results with early adopter doctors, build a training center to train new people, and then publish good results. It is also thought that the small size of the company has affected the spread of the procedure, as it was not possible to show safety and efficacy through large-scale clinical trials or to conduct a major promotional campaign.

Currently, the procedure is performed in >40 countries around the world, including the United States. We have set up training courses in various locations. We plan to expand this to make it easier for more doctors to become familiar with the procedure. We also plan to publish clinical papers on various indications in addition to the indications that are currently being performed.

**LSL: What is the process you have been using for training clinicians in the use of your system?**

**HS:** Promotion and education programs were conducted by TaeWoong Medical, our global exclusive distributor. Regular and intensive training workshops were held in South Korea, France, Costa Rica, and other countries for many users who were not familiar with the procedure. Similar programs were held in various parts of the world whenever the opportunity arose.

Users were first familiarized with the procedure and product through hands-on models that were specially created. They were then given the opportunity to gain experience through animal experiments or to understand the procedure by observing live cases.

After the COVID-19 pandemic, we also focused on online educational content through webinars and educational videos.

**LSL: What are the future plans for the RFA system? What things are you working on next?**

**HS:** We are researching products that can improve the treatment effect by optimizing the RF energy output pattern for each device and lesion characteristics. We are also working to develop products that can provide more precise treatment by using various energy sources other than RF energy that can increase patient safety and accurately treat only the target lesion area.

## Editor’s Closing Remarks

Cross-fertilization of ideas across disciplines in medicine and beyond medicine is vital to moving our field forward. Here we witness lessons from other areas of medicine that captured the imagination of a smaller company and led to furthering RFA in gastroenterology. We again observe the importance of partnership between industry and physicians to bring new technologies to market as well as a successful model of a smaller company partnering with a larger well-established gastroenterology-focused company for distribution and marketing. RFA holds promise for potential improved therapeutics in both biliary and pancreatic malignancies in which advances are critically needed. Whether RFA will provide primary and/or adjunctive treatment that prolongs survival remains unclear. Issues of improved tissue apposition with the RFA catheter within the bile duct as well as ensuring maximal tumor ablation without causing injury to normal pancreatic tissue require further refinement in the devices and techniques.

## Disclosure

The following authors disclosed financial relationships: H. K. Shin: Chief Executive Officer of STARmed Co, Ltd. L. Lee: consultant for Boston Scientific, Fujifilm Medical, and Fractyl.
